# Increased wave action promotes muscle performance but increasing temperatures cause a tenacity–endurance trade-off in intertidal snails (*Nerita atramentosa*)

**DOI:** 10.1093/conphys/coz039

**Published:** 2019-07-18

**Authors:** Samuel Clayman, Frank Seebacher

**Affiliations:** School of Life and Environmental Sciences A08, University of Sydney, New South Wales, Australia

**Keywords:** Climate change, endurance, force, metabolism, phenotypic plasticity

## Abstract

Concurrent increases in wave action and sea surface temperatures increase the physical impact on intertidal organisms and affect their physiological capacity to respond to that impact. Our aim was to determine whether wave exposure altered muscle function in intertidal snails (*Nerita atramentosa*) and whether responses to wave action and temperature are plastic, leading to compensation for altered environmental conditions. We show that field snails from exposed shores had greater endurance and vertical tenacity than snails from matched protected shores (n = 5 pairs of shores). There were no differences in muscle metabolic capacities (strombine/lactate dehydrogenase, citrate synthase and cytochrome c oxidase activities) between shore types. Maximum stress (force/foot area) produced by isolated foot muscle did not differ between shore types, but foot muscle from snails on exposed shores had greater endurance. A laboratory experiment showed that vertical tenacity was greater in animals acclimated for 3 weeks to cool winter temperatures (15 C) compared to summer temperatures (25 C), but endurance was greater in snails acclimated to 25°C. Acclimation to water flow that mimicked wave action in the field increased vertical tenacity but decreased endurance. Our data show that increased wave action elicits a training effect on muscle, but that increasing sea surface temperature can cause a trade-off between tenacity and endurance. Ocean warming would negate the beneficial increase in tenacity that could render snails more resistant to acute impacts of wave action, while promoting longer term resistance to dislodgment by waves.

## Teaser

Increased wave action may dislodge intertidal organisms, thereby reducing survival and fitness. We show that wave action can lead to improved muscle strength. However, increases in temperature can diminish the benefits by decreasing the capacity of the muscle to produce the force needed to withstand acute wave action, thereby rendering intertidal snails vulnerable to climate change when increased wave action is combined with warming.

## Introduction

Environmental change is a principal selection pressure, and the complexity of natural environments means that animals often respond to changes in more than a single environmental driver at a time (Brierley and Kingsford, 2009). The capacity to compensate for the potentially negative effects of concurrent changes in several environmental drivers therefore determines the persistence of natural populations (Rosenblatt *et al.*, 2017). For example, anthropogenic climate change has already led to increased temperatures, increased wind speed and increased wave heights in oceans (Aarnes *et al.*, 2017; Hemer et al., 2013; Young *et al.*, 2011), as well as to ocean acidification (Doney *et al.*, 2009). Increased wind speed and wave height may lead to increased physiological demand on birds (Nourani *et al.*, 2017) and marine organisms (Bejarano *et al.*, 2017; Forrester *et al.*, 2016), respectively. At the same time, temperature changes alter physiological performance (James, 2013; Olberding and Deban, 2017), thereby modifying the impact of other drivers. To understand and predict the impacts of current and future climate change therefore requires analysis of the interaction between these environmental drivers. The aim of this study was to determine whether plastic responses in muscle performance can compensate for the interaction between changing wave action and temperature in an intertidal organism (the gastropod *Nerita atramentosa*).

Intertidal organisms are an ideal study system because they are exposed to several environmental drivers concurrently. Wave action is of particular importance because it can dislodge individuals, thereby effectively removing them from the population (Jensen and Denny, 2016). One of the most important traits for survival of intertidal organisms is the adhesion strength to the substrate (Trussell *et al.*, 1993). In gastropods, adhesion strength is mediated principally by the columellar and pedal muscles (Thompson *et al.*, 1998). Adhesion strength, or tenacity (whole-animal dislodgement force per unit foot surface area, in N·cm^−2^), is influenced by foot surface area, and it depends on pedal mucus characteristics (Branch and Marsh, 1978; Iwamoto *et al.*, 2014). The foot muscle has to produce greater force for the snail to stay *in situ* as wave action increases (Forrester *et al.*, 2016), and the effect of wave impact is modulated by the size and shape of the organism (Trussell *et al.*, 1993). Force needs to be produced over longer time periods if the increase in wave action is chronic. Conceptually, therefore, wave action acts as a training regime, and snails would need to increase both short-term force production and endurance to avoid dislodgement by increased acute wave action and chronic changes in wave regimes, respectively. However, a muscle sprint-endurance trade-off may constrain compensatory responses, so that either force or endurance are maximized, but not both (James *et al.*, 1995; Wilson and James, 2004).

Additionally, both force production and endurance are affected non-linearly by temperature (Guderley *et al.*, 2009; James, 2013). Hence, the persistence of snails on rocky shores is likely to be influenced by the interactive effects of wave action and temperature on muscle performance. The effects of temperature on physiological performance such as muscle contractile function typically follow a Gaussian function (inverted ‘U’), where performance decreases at temperatures below and above the maximum (Huey and Kingsolver, 1989). The interaction between temperature and wave action on muscle performance is therefore complex, and it is important to determine this relationship empirically. We addressed our aim by comparing natural populations of snails on exposed and protected shores to test the hypothesis that (i) a training effect on muscle in response to increased wave action led to increased tenacity and endurance of snails on wave-exposed shores. Secondly, we acclimated snails from a protected shore to different temperature and water flow (mimicking wave action) regimes to test the hypotheses that (ii) muscle performance in snails is plastic and can respond positively to increased demands, and (iii) performance will decrease as temperatures exceed current mean temperatures. Alternative hypotheses are that muscle performance is not plastic and differences, if any, are due to other causes such as selection at time of larval settlement. Additionally, snails may be temperature-limited so that ‘warmer is better’ (Angilletta *et al.*, 2010), and muscle performance increases at warmer temperatures.

The intertidal snail *N. atramentosa* (Reeve) is common on all shore types along the south eastern and southern Australian coastline. Dispersal is achieved via planktonic larvae, thus minimizing the chances of genetic isolation of populations at smaller spatial scales (Teske *et al.*, 2016). The pelagic larval stage of *N. atramentosa* remains in the water column for at least 1 week (Underwood, 1974). *Nerita atramentosa* inhabits the middle to upper regions of the intertidal, foraging during immersion and for a short time after the ebb (Chapperon and Seuront, 2012), which exposes *N. atramentosa* to wave action for the majority of its active period.

## Materials and methods

### Field experiment

#### Study sites

**Figure 1:**
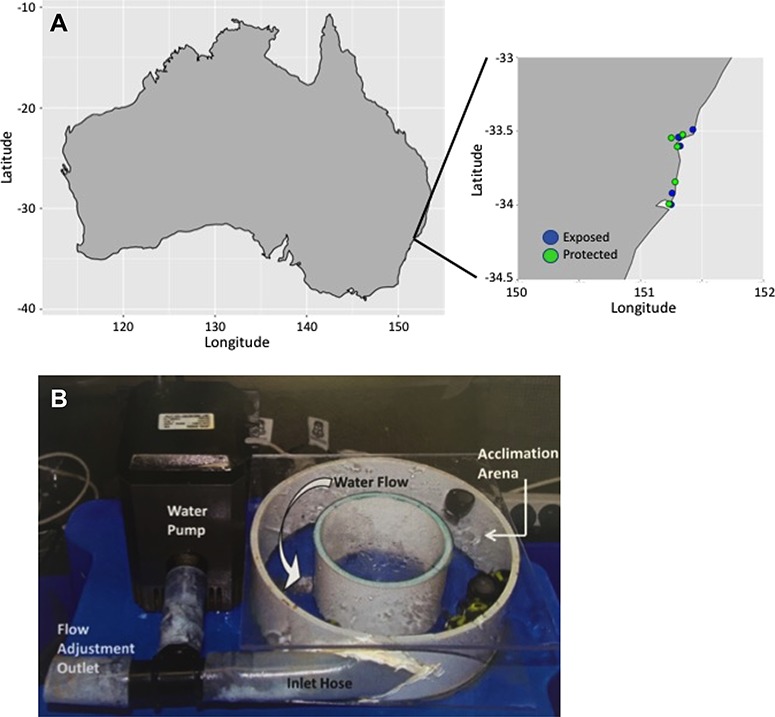
Field study sites and acclimation chamber. We chose five pairs of sites in New South Wales, Australia, and within each site there was one rocky shore exposed to the open ocean and the consequent wave action (exposed sites) and the other faced away from the ocean and was protected (protected sites) from wave action (A). Flow acclimation chamber showing the submersible water pump, the flow adjustment outlet tube, the water inlet tube and the acclimation arena within the two PVC rings. The direction of water flow is indicated by the thick white arrow. The elastic bands used to hold the lid and base together are not shown (B).

Field sampling and measurements of *N. atramentosa* endurance and tenacity were made at five sites, and within each site we paired an exposed shore with a protected shore (Fig. 1A). Exposed shores faced the open ocean with unobstructed exposure to incoming waves and swell. Protected shores were completely protected by land from ocean waves and swell. The five pairs of exposed and protected shores were selected in the following locations (listed as exposed and protected, respectively): (i) Ivo Rowe rock pool, South Coogee, NSW (33°55′08″S, 151°15′20″E﻿) and Camp Cove, Watson’s Bay, NSW (33°50′33.9″S, 151°16′52.5″E﻿); (ii) Cape Banks, Port Botany, NSW (33°59′44″S, 151°14′58″E﻿), and Bare Island, La Perouse, NSW (33°59′32″S, 151°13′52″E﻿); (iii) Green Point, Pearl Beach, NSW (33°32′31″S, 151°18′29″E﻿), and Spring Beach, Little Wobby, NSW (33°32′46″S 151°15′00″E﻿); (iv) McMasters Beach, NSW (33°29′24″S, 151°25′34″E﻿), and Half Tide Rocks, Wagstaffe, NSW (33°31′26″S, 151°20′35″E﻿); (v) Palm Beach, NSW (33°36′04″S, 151°19′18″E﻿), and Bennett’s Point, NSW (33°36′18″S, 151°17′56″E﻿) (Fig. 1A).

#### Morphology

Morphological measurements were taken from all snails sampled for field (total n = 55/site) and acclimation experiments (see below) after the dislodgement experiments. Shell height (H) and length (L) were measured with callipers to the nearest 0.1 mm (Table 1) and used to estimate the maximum projected surface area (MPSA, as H × L) of the shell, which is a standard shape measure for gastropods (Trussell *et al.*, 1993). Each snail was then placed onto a plastic petri dish with ~5 mL of seawater. The dish was tilted to 45 degrees or more until the snail stopped moving and had adhered to the dish. Adhesion was evident by a change in the shape of the foot, becoming more evenly elliptical than during crawling. Each dish had a 1 cm^2^ scale area marked, and a digital photograph was taken of the foot of each snail to determine the foot surface area; the surface area of the foot (cm^2^) was calculated in Image J software (NIH, USA) (Trussell *et al.*, 1993).

**Table 1:** Mean height and length of snails used in field and acclimation experiments

	Mean height ± s.e. (mm)	Mean length ± s.e. (mm)
**Field**		
Exposed 1	10.9 ± 0.1	18.7 ± 0.16
Protected 1	11.9 ± 0.27	20.2 ± 0.41
Exposed 2	11.0 ± 0.1	18.8 ± 0.3
Protected 2	10.6 ± 0.2	18.0 ± 0.3
Exposed 3	11.0 ± 0.2	19.2 ± 0.3
Protected 3	12.4 ± 0.2	22.2 ± 0.3
Exposed 4	12.1 ± 0.1	20.3 ± 0.2
Protected 4	11.0 ± 0.2	19.5 ± 0.4
Exposed 5	10.2 ± 0.2	18.2 ± 0.3
Protected 5	11.0 ± 0.3	18.6 ± 0.4
**Acclimation**		
15S	11.8 ± 0.09	19.8 ± 0.1
15F	11.6 ± 0.08	19.5 ± 0.1
25S	11.3 ± 0.09	18.8 ± 0.01
25F	11.5 ± 0.07	19.1 ± 0.1

Exposed, exposed shores; protected, protected shores (numbering as in main text); 15S, 15 C acclimation and still water; 15F, 15 C acclimation and flowing water; 25S, 25 C acclimation and still water; 25F, 25 C acclimation and flowing water.

#### Dislodgement forces and endurance

On each shore, individual *N. atramentosa* were randomly selected for measurements of acute vertical (n = 20/shore) and horizontal dislodgement force (n = 20/shore). Different snails were used for vertical and horizontal measurements. Vertical dislodgement force was defined as the force necessary to dislodge the animal in a direction perpendicular to the substrate. Horizontal dislodgement force was defined as the force necessary to dislodge an animal in a direction parallel to the substrate. A rectangular piece of aluminium mesh (~10 × 40 mm) was folded into a ‘T’ shape (herein referred to as ‘mesh tabs’). The tab was inverted, and the flat top of the ‘T’ was attached with cyanoacrylic glue to either the top of the shell to test vertical dislodgement force or the rear of the shell to test horizontal dislodgement force. Only snails on a relatively flat surface that was free of macroalgae were sampled. All samples were obtained from snails on wet sandstone substrate, which is the predominant type of rock in the region. Measurements were obtained only from animals *in situ*, and none were moved by hand to suitable sampling areas.

A force transducer (model MLT1030/A, AD Instruments, Australia), connected to a computerized recording system (Powerlab 4/20, AD Instruments, Australia) via a bridge amplifier (model ML301, AD Instruments, Australia), was used to measure the vertical and horizontal dislodgement forces; we adjusted the PowerLab and recording system for field use by using a 12-volt battery and a power inverter. A small hook was tied to the force transducer with nylon line, and the hook was slipped through the mesh tab on the back of a snail. The string was slowly pulled taut, and the snail was softly tapped on the shell with a pen to induce adhesion (Branch and Marsh, 1978). Upon full adhesion, the transducer was moved away manually at a rate such that the snail was dislodged within 1 s (estimated with a stopwatch).

Endurance was measured as the time to dislodgement after applying a constant vertical force to individual *N. atramentosa* (*n* = 15/shore). To apply the force, we used a pulley system, consisting of a stand (height: 250 mm) with a horizontal arm (length: 100 mm) supporting a pulley wheel (diameter: 25 mm). Nylon fishing line with a hook at one end and lead weights (80 g) on the other was looped over the wheel; the hook was attached to dorsal mesh tabs as described above, and the lead weights applied a constant force of 0.78 N. This weight was determined during pilot tests to ensure that each snail could resist the weight for at least 2 min. Endurance was measured as the time from application of the full force until the snail was dislodged from the substrate.

#### Enzyme activities

We determined (in 10 snails per shore) aerobic metabolic capacity as activities of cytochrome c oxidase (COX) and citrate synthase (CS), and the capacity for anaerobic energy production as combined strombine/lactate dehydrogenase (LDH) activity. In gastropods, a family of glycolytic pyruvate reductase enzymes called opine dehydrogenases (ODHs) function in the same metabolic pathway as LDH, and thus the maximal anaerobic capacity can be estimated by their combined activities (Sato *et al.*, 2013). Snails used for enzyme assays were not used for any other measures.

All assays were conducted in duplicate at 20°C, which was the mean water temperature during the collection period. Foot muscle tissue was excised and instantly frozen in liquid nitrogen and then stored at −80°C for later analysis. For the assays, tissue (0.06–0.08 g/sample) was homogenized in nine volumes of extraction buffer (50 mM imidazole/HCl, 2 mM MgCl_2_, 5 mM ethylene diamine tetra-acetic acid (EDTA), 1 mM reduced glutathione and 0.1% Triton X-100, pH 7.5) (Sinclair *et al.*, 2006). The homogenate was centrifuged for 4 min and 10 μL of the supernatant was added to 990 μL of assay medium for each assay.

For the COX assay, a reference of 0.05 mM cytochrome c oxidized with 50 mM potassium ferricyanide (K_2_F(CN)_6_), was used and the assay medium contained 100 mM KH_2_PO_4_/K_2_PO_4_ (pH 7.5) and 0.05 mM cytochrome c; cytochrome c in the assay medium was reduced by adding sodium hydrosulphide (Na_2_SO_4_) and bubbling with air to remove excess sodium hydrosulphide. CS activity was assayed in 100 mM Tris/HCl (pH 8.0), 0.1 mM DTNB, 0.1 mM acetyl CoA and 0.15 mM oxaloacetate. Control assays without oxaloacetate were conducted for each sample.

The combined activity of LDH and strombine dehydrogenase (SDH) was assayed in 100 mM potassium phosphate (KH_2_PO_4_/K_2_PO_4_) buffer (pH 7.0), with 0.16 mM NADH, 1 mM pyruvate and 100 mM glycine. Both enzymes require NADH, while SDH also requires the amino acid glycine to catalyse the forward reaction, and the single assay gives an estimate of combined anaerobic metabolic capacity in the foot muscle (Sato *et al.*, 2013; Sinclair *et al.*, 2006). We chose LDH and SDH to estimate anaerobic metabolism based on pilot studies in which we assayed the relative contributions of all ODHs (strombine, octopine, tauropine and alanopine; Sato et al. 2013). Activity of each enzyme was determined by tracking the change in absorbance over time of reduced cytochrome *c* at 550 nm (COX), of DTNB at 412 nm (CS) or of NADH at 340 nm (LDH/SDH) in a UV/visible spectrophotometer (Ultrospec 2100 pro, Biochrom, UK) with a temperature controlled cuvette holder.

#### Muscle mechanics

We measured the mechanics of isolated muscle to determine whether wave impact affected contractile function of isolated muscle. We dissected the whole foot muscle from individual *N. atramentosa* from Palm Beach (*n* = 20; exposed) and Bennett’s Point (*n* = 20; protected) only. Snails from Palm Beach and Bennett’s Point were sampled alternately throughout the experiment to avoid order effects, and snails were used within 36 h of collection and were held in aerated seawater while in the laboratory.

Prior to dissection, the shell height and length were measured with callipers, and a digital photograph was taken of the foot, as above. Dissections were performed by cutting the shell with a diamond-dust disc bit (~20 × 1.5 mm) attached to a high-speed dental drill (Flexible Shaft Motor, Faro, Italy). The foot muscle of *N. atramentosa* has two attachment points at the interior of the shell. Each covers an area of ~2 × 4 mm, and they are located roughly at opposite ends of the long axis from the operculum to the back of the shell. The terminal end of the muscle attaches along the posterior edge of the operculum and its spur. The entire snail was excised from the shell with the operculum and two rectangular tabs of shell remaining (~4 × 7 mm) at the muscle attachment regions. The visceral mass, organs, mantle and head were removed before mounting the preparation.

Isolated muscle force measurements were made in a Perspex organ bath (190 × 35 × 40 mm) filled with gastropod Ringer solution (Olson and Marsh, 1993) and held at 20°C (± 0.5) throughout sampling. The Ringer solution was changed after every two samples. A force transducer (MLT1030/A, AD Instruments, Australia) was attached to a computerized recording system (Powerlab model 4/20, AD Instruments, Australia) via a bridge amplifier (ML301, AD Instruments, Australia) and held vertically with the end suspended in the Ringer solution. An alligator clip (32 mm) was attached to the force transducer, to which one of the shell tabs of the dissected muscle was attached. At the other end of the chamber, a modified microscope table was fixed to the bench and fitted with a second alligator clip extending into the Ringer solution, to which the tab at the other end of the muscle preparation was attached.

In preliminary experiments, we determined that the smooth muscle of the snail responded better to vibration rather than to electrical stimuli. Hence, we designed an apparatus that delivered vibrations at a constant rate. We fitted a wire arm with a lead weight (80 g) attached to its terminal end to the axle of a small electrical motor (Duratech 12 V, Jaycar Electronics, Australia). The motor dropped the weight on the bench with each rotation, thereby eliciting a stimulus for muscle contraction. The motor was powered by a variable DC power supply (NP-9615, Manson Engineering Industrial, Hong Kong, China), which was adjusted so that the rotation speed was 40 rpm.

Before sampling, we determined the muscle length that produced the greatest force of contraction for each preparation. After the muscle was attached to the alligator clips in the Ringer solution and gently extended, it was rested for 4 min. We then provided a single stimulus and measured contractile force, after which the muscle was stretched by 1 mm and allowed to rest for 4 min before the next stimulus. This process was repeated five times, and for experiments we set the muscle length to coincide with the greatest force produced. The muscle was left to rest at that length for 5 min before starting the motor-driven stimulus. The motor was left to run for 60 min, after which it was turned off for 5 min with the muscle still attached. The motor was then briefly switched on to stimulate a single contraction as verification that the muscle was still functioning. After sampling, the tabs of shell and the operculum were removed from the muscle preparation, which was patted dry and weighed to the nearest 0.001 g. We recorded maximum stress (N cm^−2^ of muscle cross-sectional area), and the number of contractions greater than 80% of maximum and 50% greater than maximum as an estimate of endurance during each trial.

### Acclimation experiment

#### Collection site

Snails (n = 200) of similar size were collected from a protected shore (Bennett’s Point). We measured water velocities (with a flow meter: Model FP101, Global Water, USA) at Bennett’s Point and a nearby wave-exposed site (Palm Beach) on the same day during mild conditions with moderate wave action. Velocities (0.12 ± 0.005 [s.e.] m/s at Bennett’s Point, 0.43 ± 0.027 [s.e.] m/s at Palm Beach) were used to calibrate the experimental water flow treatments (see below). We also collected rocks from Bennett’s Point for use as a microalgae food source during the experiment.

#### Acclimation treatments and animal husbandry

We designed and constructed flow-through acclimation chambers (20 chambers; Fig. 1B) that allowed us to alter water flow and temperature simultaneously. The chambers were constructed of a section of PVC pipe (160 mm diameter × 3 mm thickness × 40 mm width) glued to the centre of a clear PVC sheet (165 × 165 × 3 mm) to form the base. A similar part consisting of a smaller PVC pipe (80 × 3 × 40 mm) glued to the centre of a second clear PVC sheet (165 × 165 × 3 mm) formed the lid. The two parts were assembled so that the smaller PVC pipe was situated at the centre of the larger one, forming an annulus. We used two heavy-duty elastic bands to hold the two separate parts of the chamber together firmly during treatments.

We fitted a flexible plastic hose (25 mm diameter) into an oblong aperture cut into the larger PVC pipe so that it was flush with the inner wall of the pipe. The hose served as a water inlet. The distal end of the hose was fitted with a T-connector, to which a submersible pump (Model JHQ 2000, J&T Industry and Co., China) was connected at one opening, and the diameter of the second opening could be altered to adjust the water flow into the chamber. In the flow treatment, we adjusted water flow to 0.40–0.45 m/s within the chamber arena, which corresponded to measurements of water flow at the exposed shore. We determined water flow inside the chambers by filming particles moving in the chambers and analysing the videos in Tracker Video Analysis and Modeling software (https://physlets.org/tracker/). There was no water flow in the still-water treatments.

Before assembly, we placed snails into the annulus for acclimation treatments. Ten snails were randomly assigned to each of the 20 acclimation chambers, and 10 chambers were kept in each of two temperature-controlled rooms, set to 15 and 25°C, for thermal acclimation treatments. Total acclimation period was 21 days for both water flow and temperature. Within each acclimation temperature treatment there were five flow-treatment chambers (denoted 15F and 25F) and five still-water treatment chambers (15S and 25S) (*n* = 50 snails/treatment group). Each chamber was placed into a plastic tank with 8 L of aerated seawater, and the light cycle was 12 h light:12 h dark. For 8 h each day (4 h in light and 4 h in dark) the chambers were removed from the water, drained, and a food rock was placed into the chamber for the full 8 h. We did not leave food rocks in the tanks when water flow was present because the rocks would have blocked the flow, and snails feed readily when exposed. Between feeding periods, food rocks were kept in a separate bin with aerated, circulating seawater, and new rocks were collected during the experiment. Of the 16 h spent in water, 8 h were in light and 8 h were in darkness. For the flow regime, the water pumps were set to alternate between 1 h on and 1 h off for the duration of the period in the water.

We collected body temperature data in the field at Pearl Beach, NSW (33°32′31″S, 151°18′29″E), in winter (July) and summer (January) to inform acclimation temperatures. Randomly selected snails were sampled 1, 3 and 6 h after high tide on each of three days and both at night (n = 58 snails in winter and 91 snails in summer) and during the day (n = 149 snails in winter and 145 snails in summer). Body temperatures were measured by inserting a hand-held K-type thermocouple (Model C2000, Cope Digital Thermometers, London, UK) into the shell. Mean (± s.e.) body temperature in winter were 15.6 (± 0.2)^o^C during the day and 9.8 (± 0.3)^o^C at night. Summer body temperatures were 25.1 (± 0.2)^o^C during the day and 21.2 (± 0.1)^o^C at night. Corresponding water temperatures were 15.8 (range 15.2–16.7)^o^C in winter and 22.4 (range 21.2–23.2)^o^C in summer.

#### Dislodgement force and endurance measures

After the acclimation period, we measured vertical dislodgement and endurance as described above for field experiments, on smooth-surfaced sandstone paving stones (200 × 200 × 30 mm) wetted with fresh seawater to mimic the natural sandstone substrate. Measurements were made at 15 and 25°C acute test temperatures. Four snails were randomly chosen from each of the five chambers within each of the four treatments for dislodgement force measurements (n = 20/treatment), and three different snails were randomly chosen from each chamber for endurance measurements (n = 15/treatment). Snails were placed on the paving stones and allowed 5 min to crawl freely before sampling. Each individual was tested first at its acclimation temperature, allowed to recover for 36 h, then tested at the other acclimation temperature. Snails were allowed to equilibrate to the second test temperature for 1–3 h before measurements. During the 36 h recovery period, the snails continued the daily rotation of feeding and underwater periods. We measured foot area after the acclimation period as described for field experiments above.

#### Statistical analysis

We analysed all data with permutational analyses of variance. We chose permutational tests because the analysis uses the data per se rather than making assumptions about underlying distributions (Drummond and Vowler, 2012). Analyses were conducted in the lmPerm package in R (Wheeler and Torchiano, 2016). Field data (endurance, tenacity, foot area, MPSA and enzyme activities) were analysed with shore type (protected or exposed) as fixed factor and site nested within shore type. In all analyses of endurance we used foot area as a covariate. We compared muscle mechanics data (maximum stress [force/muscle cross-sectional area], number of peaks above 80% of maximum and number of peaks above 50% of maximum) between snails collected from a protected and a exposed shore. Data from the acclimation experiment (tenacity, endurance) were analysed with wave impact (present or absent), acclimation temperature (15 or 25°C) and test temperature (15 or 25 C) as factors. The truncated product method showed that multiple hypotheses tests did not affect the validity of *P*-values (Zaykin et al. 2002). All means ± s.e. are given, and we report permutational probabilities.

## Results

### Field data

**Figure 2:**
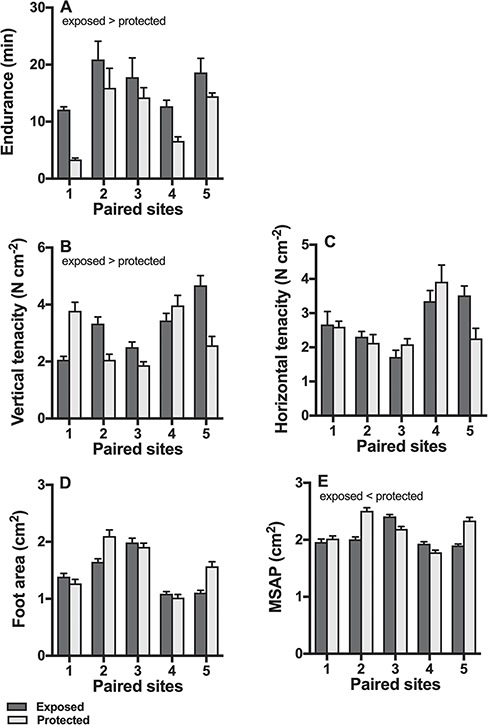
Field endurance, tenacity and morphology. Snails from exposed shores (dark bars) had greater endurance than those from protected sites (light bars), but there were significant differences between sites (A, n = 15 snails per site). Similarly, the vertical tenacity was greater in snails from exposed shores, but there were significant differences between sites (B; n = 20 per site). Shore type had no effect on horizontal tenacity (C, n = 20 per site) or on foot size (D, n = 55 per site), but snails from exposed shores had smaller MSPA compared to snails from protected shores (E, n = 55 per site); in all cases, there were significant differences between sites. Means ± s.e. are shown.

Endurance was significantly greater in snails from exposed sites compared to protected sites (*P* < 0.001; Fig. 2A), but there were significant differences between sites (shore type × site interaction *P* = 0.0058). Endurance increased significantly with foot area (*P* = 0.0088).

Vertical tenacity was greater in snails from exposed sites (*P* = 0.024; Fig. 2B), but there were significant differences between sites (shore type × site interaction *P* < 0.0001). Horizontal tenacity was not affected by shore type (*P* = 0.36; Fig. 2C), but it differed significantly between sites (shore type × site interaction *P* < 0.001).

Foot area did not differ between exposed and protected shores (*P* = 0.14; Fig. 2D), but it differed significantly between sites (shore type × site interaction *P* < 0.001). MPSA was greater in snails from protected shores (*P* = 0.010; Fig. 2E), but there were significant differences between sites (shore type × site interaction *P* < 0.001).

**Figure 3:**
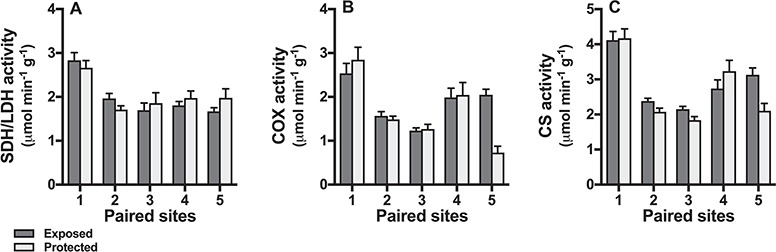
Enzyme activities of field-collected snails. Shore type (exposed = dark bars, protected = light bars) has no effect on octopine/LDH (A), COX (B) or CS (C) activities, but in all cases there were significant differences between sites. Means ± s.e. are shown and n = 10 snails per shore.

Activities of strombine/LDH, CS, and COX did not differ between snails from exposed and protected shores (all p > 0.15; Fig. 3), but in all cases there were significant differences between sites (all shore type x site interactions *P* < 0.001).

### Muscle mechanics

**Figure 4:**
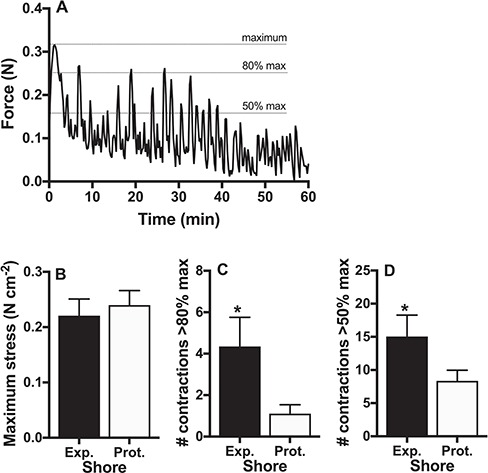
Mechanics of isolated muscle. An typical experimental trace (A) shows maximum force production, and 80% and 50% of maximum, which we used to evaluate endurance. Maximum stress (B) did not differ between snails from exposed (Exp., black bars) and protected shores (Prot., white bars), but snails from exposed shores showed greater fatigue resistance and produced more contractions above 80% of maximum (C) and above 50% of maximum (D) over time, compared to snails from protected shores. Means ± s.e. are shown and n = 20 snails per shore from a single site; asterisks indicate significant differences between shores.

A typical trace from one experimental run is shown in Fig. 4A. There were no differences between snails from exposed and protected shores in the maximum stress (force/foot area) produced by their isolated foot muscle (*P* = 0.53; Fig. 4B). However, snails from exposed areas maintained greater force for longer time, such that both the number of contractions above 80% of maximum stress (*P* = 0.011; Fig. 4C) and the number of contractions above 50% of maximum stress (*P* = 0.032; Fig. 4D) were higher in snails from the exposed shore.

### Acclimation experiment

**Figure 5:**
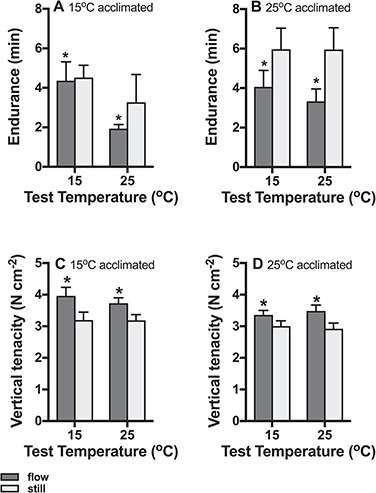
Responses to acclimation to wave impact and temperature. Endurance was greater in the 25°C acclimation treatment compared to the 15°C treatment, but it decreased significantly with water flow compared to still water (A, B). Vertical tenacity increased following acclimation to water flow in both 15°C (C) and 25°C (D) acclimated snails. Test temperature had no effect on tenacity, but 15°C acclimated snails had greater tenacity than those acclimated to 25°C. Means ± s.e. are shown and n = 20 snails per treatment group.

Endurance differed significantly between acclimation treatments (*P* = 0.043), and it was greater in the 25°C acclimated animals compared to the 15°C acclimation treatment (Fig. 5A, B). Water flow decreased endurance significantly (*P* = 0.044; Fig. 5). Test temperature and all interaction did not have a significant effect (all *P* > 0.14; Fig. 5A, B).

Vertical tenacity was significantly greater in snails acclimated to water flow compared to animals in still water (*P* < 0.0001; Fig. 5C, D), and it was greater in 15°C acclimated animals compared to those acclimated to 25°C (*P* = 0.017; Fig. 5C, D). There was no effect of test temperature (*P* = 0.39), and there were no significant interactions (all interactions *P* > 0.37).

## Discussion

We have shown that wave action alters the capacity of intertidal gastropods to adhere to their substrate. This effect is mediated partly by muscle plasticity, and wave action represents a training regime that increases the tenacity of snails. Interestingly, endurance of snails decreased with increased exposure to wave action in the laboratory, so that the increased endurance we observed on exposed shores in the field is unlikely to be mediated by acclimation although it may be have been modified by developmental conditions (Beaman et al. 2016). It is possible also that selection for high endurance phenotypes, either at the time of settlement or subsequently, caused differences between protected and exposed shores. The combined impacts of wave exposure and temperature on performance of *N. atramentosa* mean that warming sea temperatures will lead it to face a trade-off between tenacity and endurance, with decreased capacity to withstand the acute impacts of waves but improved long-term persistence.

The rocky intertidal region is among the most variable habitats on earth, with tides producing twice-daily aquatic submersion and exposure to atmospheric conditions. Temperatures and wave energies fluctuate daily and seasonally, and wave energies also differ between shores depending on the degree of exposure to open ocean swells (Helmuth *et al.*, 2006; Jensen and Denny, 2016). Additionally, both temperature and wave action are increasing with anthropogenic climate change (Pecl *et al.*, 2017; Semedo *et al.*, 2011). Lift and drag forces imparted by waves can dislodge rocky shore inhabitants (Denny and Blanchette, 2000; Forrester *et al.*, 2016), so that muscle performance may have a direct influence on the survival of individuals within the wave-swept intertidal region. Temperature has profound effects on physiology in general and muscle performance specifically (Drake *et al.*, 2017; James, 2013). Hence, thermodynamic effects on muscle and plastic responses by muscle to altered temperatures (acclimation) and wave action (training) will have profound effects on the fitness of intertidal organisms. Widely distributed intertidal species can include populations that experience different chronic thermal and hydrodynamic conditions so that populations within species may be affected differentially by climate change.

Gastropod snails are a conspicuous group of intertidal organisms, inhabiting virtually every microhabitat of wave-swept intertidal gradients around the world. Many gastropod species are distributed across high- and low-energy shores, which can influence intraspecific morphology (Forrester *et al.*, 2016; Solas *et al.*, 2015). For example, shells of exposed-shore populations are typically smaller and thinner, with larger foot surface areas when compared to protected-shore conspecifics (Trussell, 1997). We found that on average surface area was smaller in snails from exposed shores, and the resulting decrease in drag may aid physiological mechanisms to counteract the greater dislodgement forces experienced on exposed shores. Greater dislodgement forces are also associated with increased stiffness of the pedal musculature (Branch and Marsh, 1978), possibly increasing tenacity without a concomitant increase in overall foot size.

Locomotion in many gastropods is achieved by a succession of muscular waves generated by alternate contraction and relaxation of the foot muscle. The force generated by the foot muscle is coupled to the substrate via the layer of pedal mucus (Iwamoto *et al.*, 2014). Gastropod muscle is typically comprised of striated muscle that mediates phasic contractions of high force but low fatigue resistance and smooth muscle producing tonic contractions of low force and high fatigue resistance (Fleury *et al.*, 2005; Marsh and Olson, 1994; Sulbarán *et al.*, 2015). The different responses we observed in tenacity and endurance may indicate that both cannot be maximized at the same time (Wilson and James, 2004), at least not in response to relatively short term acclimation. Both acclimation to temperature and wave action elicited opposing responses in tenacity and endurance: wave action increased tenacity but decreased endurance and increased temperature reduced tenacity but increased endurance. The decrease in endurance with increased water flow in our acclimation experiment is somewhat surprising. The flow regime may not have been sufficient to stimulate endurance training. It is possible as well that energy limitation may have constrained endurance. Our field enzyme data indicate that maximal metabolic capacities do not constrain greater endurance on exposed shores. However, acute energetic status, such as phosphoarginine concentrations and adenylate charges, can affect muscle performance (Pérez *et al.*, 2008), which may at least partly explain the results of our acclimation treatments. Note also that endurance in the acclimation study was overall lower than in the field, and it is possible that time in captivity reduced muscle performance, but this effect should have been similar in all treatments.

Our data show that intertidal snails can respond positively to increased wave action in the field. Increased tenacity would improve resistance to dislodgement by acute high wave impact as a result of storms, for example, while increased endurance would be beneficial for chronic increases in wave action. However, the interactions between wave action and temperature show that changes in more than one environmental parameter can modify otherwise beneficial phenotypic responses. For example, ocean warming would negate the beneficial increase in tenacity resulting from increased wave action while promoting longer term resistance to dislodgment by waves. Predicting the effects of climate change on intertidal organisms is further complicated by differences between sites, indicating that there is substantial within-species variation. *Nerita* disperse via planktonic larvae and there is little genetic structuring at the regional level (Teske *et al.*, 2016). Phenotypic differences between sites or populations may arise from epigenetic mechanisms or from local selection at or after settlement. It would be important to determine the role of these different mechanisms to improve predictive power of the effects of future environmental change.
